# HIV‐1 Genetic Diversity and Drug Resistance Mutations Among Patients on Antiretroviral Therapy in Nairobi County, Kenya

**DOI:** 10.1155/av/3854108

**Published:** 2026-06-12

**Authors:** Erickson Angiro, Musa Ngayo, Caroline Wahome, Juster Mungiria, Anthony Nyamache

**Affiliations:** ^1^ Department of Biochemistry, Microbiology and Biotechnology, Kenyatta University, Nairobi, Kenya, ku.ac.ke; ^2^ Centre for Microbiology Research, Kenya Medical Research Institute (KEMRI), Nairobi, Kenya, kemri.org; ^3^ Department of Civil and Environmental Engineering, University of Strathclyde, Glasgow, Scotland, UK, strath.ac.uk; ^4^ Department of Biological Sciences, Chuka University, Chuka, Kenya, chuka.ac.ke

## Abstract

Kenya, a country with a significant number of human immunodeficiency virus type 1 (HIV‐1) infected individuals, has adopted the World Health Organization’s (WHO’s) “test and treat all” strategy to reduce HIV‐related deaths and new infections, aligning with the United Nations’ Acquired Immune Deficiency Syndrome (UNAIDS) 95‐95‐95 targets. In 2019, Kenya adopted the WHO’s first‐line antiretroviral Therapy (ART) recommendations, which include tenofovir/lamivudine/dolutegravir (TDF + 3TC + DTG) as a cost‐effective regimen. Despite these efforts, new HIV infections and AIDS‐related mortality continue in developing countries. This cross‐sectional study investigated HIV‐1 drug resistance mutations and subtypes among patients receiving ART in Nairobi County, Kenya. The study enrolled 227 HIV‐1‐infected patients for 12 months, with 42 out of the total 227 samples successfully amplified by polymerase chain reaction (PCR). HIV‐1 genotypic drug resistance was determined using an in‐house sequencing method. Sociodemographic data were analyzed using descriptive statistics to summarize and describe the key characteristics of the dataset. The Stanford University HIV Drug Resistance algorithm was used to analyze drug resistance mutations, and phylogenetic analysis with MEGA 11 software assessed HIV‐1 genetic diversity. The study identified key HIV‐1 drug resistance mutations, including M184V (13.73%), K103N (9.8%), Y181C (4.9%), T215Y (4.9%), and D67N (4.9%). Phylogenetic analysis of the 42 sequences generated from the study demonstrated significant genetic diversity, with Subtype A being the most dominant at 69%, followed by Subtype D (23.8%), Subtype C (4.8%), and CRF10_CD (2.4%). This study provides crucial insights into the distribution of HIV‐1 genotypes and the incidence of drug‐resistance mutations among HIV‐1 patients in Nairobi. These findings provide critical insights to guide policymakers and healthcare providers in optimizing the management and treatment of HIV‐1 patients undergoing ART.

## 1. Introduction

The human immunodeficiency virus/acquired immunodeficiency syndrome (HIV/AIDS) epidemic remains a major public health challenge globally, particularly in sub‐Saharan Africa. In Kenya, approximately 22,000 adults and children died from AIDS‐related illnesses in 2021, representing a substantial decline from approximately 64,000 deaths reported in 2010 due to expanded access to antiretroviral therapy (ART) and improved HIV care services [1]. By 2021, approximately 78% of people living with HIV in Kenya were receiving ART, while 68% had achieved viral suppression [1].

Kenya has continued to scale up ART implementation in alignment with the Joint United Nations Programme on HIV/AIDS (UNAIDS) 95‐95‐95 targets. Current treatment guidelines recommend first‐line ART regimens consisting of two nucleoside reverse transcriptase inhibitors (NRTIs) combined with an integrase inhibitor, particularly dolutegravir (DTG), in combination with tenofovir disoproxil fumarate (TDF) and lamivudine (3TC) [2]. Despite these advances, HIV‐1 drug resistance remains an important challenge that may compromise long‐term treatment success [3].

Drug resistance mutations may emerge due to prolonged drug exposure, poor adherence, viral replication under selective drug pressure, and transmitted resistant strains [4]. The emergence and transmission of resistant HIV‐1 variants may reduce treatment efficacy, increase the risk of virologic failure, and limit future therapeutic options.

Genotypic drug resistance testing has become an important tool for identifying resistance‐associated mutations and guiding treatment decisions. Compared with phenotypic assays, genotypic methods are generally more accessible, less costly, and faster to perform in resource‐limited settings [5]. In addition, HIV‐1 exhibits substantial genetic diversity resulting from rapid replication, mutation and recombination events, which may influence disease progression, transmission dynamics, and treatment response [6].

Although several studies have described HIV‐1 diversity and resistance patterns in Kenya, limited data are available on patients receiving ART in Nairobi County following expanded DTG‐based regimen implementation. Therefore, this study investigated HIV‐1 drug resistance mutations and genetic diversity among patients receiving ART in Nairobi County, Kenya.

## 2. Materials and Methods

### 2.1. Study Site

This study was conducted at the Kenya Medical Research Institute (KEMRI) in Nairobi, Kenya. The study subjects were recruited from a proportion of the cohort population attending the HIV care and treatment at the Family AIDS Care and Education Services (FACES) program, which was recently renamed the Centre for International Health, Education, and Biosecurity (CIHEB). A cross‐sectional design approach was used to enroll 227 participants following Fisher’s formula [7]. Inclusion criteria included adult patients aged 18 years and above who were on antiretroviral treatment for not less than 12 months and consented to participate.

### 2.2. Sample Collection

From each participant enrolled, approximately 5 mL of blood was collected in EDTA tubes and then centrifuged to separate the plasma. The plasma samples were then shipped in a cool box to the KEMRI HIV Laboratory, where they were stored at −80°C until ready for RNA extraction and drug resistance testing. A structured questionnaire was used to gather patients’ sociodemographic characteristics.

### 2.3. RNA Extraction

RNA was extracted from plasma samples using the QIAamp Viral RNA Mini Kit (Qiagen, USA) according to the manufacturer’s instructions with minor modifications. Briefly, plasma samples were lysed with AVL buffer containing carrier RNA, and then incubated at room temperature. Absolute ethanol was added to facilitate RNA binding to the QIAamp Mini spin column. The lysate was loaded onto the column and centrifuged, after which sequential washes with buffers AW1 and AW2 were performed to remove impurities. Viral RNA was then eluted using buffer AVE, collected into sterile microcentrifuge tubes, and stored at −80°C before reverse transcriptase polymerase chain reaction (RT‐PCR) amplification [8].

### 2.4. Amplification by RT‐PCR

The partial HIV‐1 pol RT gene, corresponding to 2265–3180 of HIV‐1 HXB2 (645 base pairs), was amplified using RT‐PCR with specific primers RT18 (5′GGAAACCAAAAATGATAGGGGGAATTGGAGG3′) and RT21 (5′CTGTATTTCTGCTATTAAGTCTTTTGATGGG3′) for the first round PCR and RT1 (5′CCAAAAGTTAAACAATGGCCATTGACAGA3′) and RT4 (5′AGTTCATAACCCATCCAAAG3′) for the nested PCR. First round PCR conditions included denaturation in 1 cycle of 45°C for 1 min and 94°C for 2 min, followed by 35 cycles of 94°C for 30 s, annealing at 55°C for 30 s, and an extension at 72°C for 10 min, with a final extension of 72°CZ. Nested PCR conditions included denaturation in 1 cycle of 94°C for 2 min, followed by 30 cycles of 94°C for 30 s, annealing at 55°C for 30 s, and extension at 72°C for 1 min, followed by a final extension at 72°C for 10 min [9].

### 2.5. Gel Electrophoresis

Amplified PCR products were analyzed using 2% agarose gel electrophoresis stained with ethidium bromide. The gel was prepared by dissolving agarose in 1X TBE buffer, followed by heating and cooling before the addition of ethidium bromide. PCR products were mixed with Orange G loading dye and loaded into the gel wells alongside HyperLadder I molecular weight markers. Electrophoresis was conducted at 90 V for approximately 30 min to allow separation of DNA fragments based on size. The amplified DNA bands were subsequently visualized under ultraviolet illumination to confirm the presence and expected size of the PCR products [10].

### 2.6. Sequencing

The confirmed amplified RT gene was cleaned using ExoSAP‐IT PCR technology to remove debris or dirty or excess primers according to the manufacturer’s instructions before sequencing. The PCR products were directly sequenced using Sanger technology according to the manufacturer’s protocol (Applied Biosystems, Foster City, CA).

### 2.7. Genotypic Drug Resistance Analysis

HIV‐1 drug resistance was determined using Stanford University’s HIV‐1 drug resistance database and interpreted according to the International AIDS Society guidelines [6]. Genotypic resistance was defined as the presence of drug resistance mutations associated with impaired drug susceptibility [11].

### 2.8. Phylogenetic Analysis

Of the 227 samples processed, 42 were successfully amplified by PCR and subsequently sequenced. These sequences, along with HIV‐1 reference sequences obtained from the Los Alamos HIV database, were analyzed using MEGA Software Version 11. Sequence alignment was performed using ClustalW, and evolutionary distances were inferred using the Neighbor‐Joining method. A phylogenetic tree was constructed using the maximum likelihood approach, with reliability assessed through 1000 bootstrap replications. The resulting tree was visualized using TreeView PPC version 1.6.6. Genotyping was performed using the REGA HIV‐1 Subtyping Tool Version 3.0.

### 2.9. Ethical Consideration

The study protocol and informed consent form were reviewed and approved by the Kenyatta University Ethical Review Committee (Application number: PKU/873/1937). Informed consent was obtained from all participants before any procedures were performed. Participant information was kept confidential, and participation was voluntary, with the option to withdraw at any time.

## 3. Results

### 3.1. Participants’ Sociodemographic Characteristics

This study enrolled 227 participants, including 130 females (57.3%) and 97 males (42.7%). The majorities were married (69.2%), followed by single (25.6%), divorced (4.8%), and widowed (0.4%). Regarding occupation, 45.4% were self‐employed, 35.2% employed, and 19.4% unemployed. Regarding education, 37% had tertiary education, 33.9% secondary, and 29.1% primary. The majorities of participants were nonsmokers (94.7%) and had never consumed alcohol (81.5%), as presented in Table 1.

**TABLE 1:** Sociodemographic features of study participants.

Variable	Frequency	Percentage (%)
Gender		
Female	130	57.3
Male	97	42.7
Marital Status		
Divorced	11	4.8
Married	157	69.2
Single	58	25.6
Widowed	1	0.4
Occupation		
Employed	80	35.2
Self‐employed	103	45.4
Unemployed	44	19.4
Education		
Primary	66	29.1
Secondary	77	33.9
Tertiary	84	37
Smoking		
Unanswered	3	1.3
No	215	94.7
Yes	9	4
Drinking		
Never	185	81.5
Occasionally	41	18.1
Regularly	1	0.4

### 3.2. HIV‐1 Drug Resistance

**FIGURE 1:**
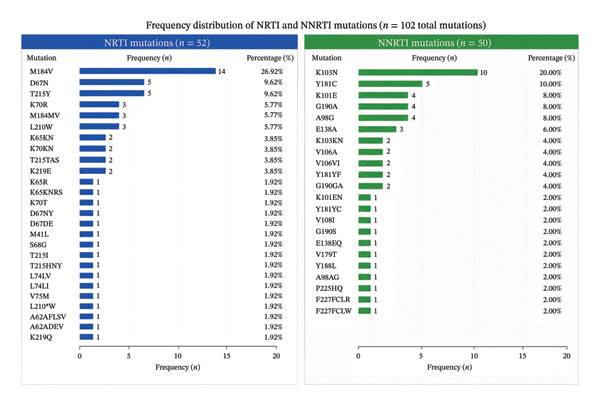
Frequency distribution of NRTI and NNRTI mutations.

Out of the 42 samples sequenced, 29 (69.05%) were infected with drug‐resistant strains while 13 (30.95%) were susceptible (wild type). The rest of the samples (185) failed to amplify by the RT‐PCR due to a low viral load (< 1000 copies/mL). The drug resistance mutations were categorized into NRTIs and non‐NRTIs (NNRTIs). Of the 102 detected mutations, 52 (50.98%) were NRTIs and 50 (49.02%) were NNRTIs. The most dominant mutations were M184V (13.73%), K103N (9.8%), Y181C (4.9%), T215Y (4.9%), and D67N (4.9%). Other notable mutations included K101E (3.92%), G190A (3.92%), A98G (3.92%), K70R (2.94%), M184MV (2.94%), L210W (2.94%), and E138A (2.94%), as presented in Figure 1.

### 3.3. HIV‐1 Genetic Diversity

**FIGURE 2:**
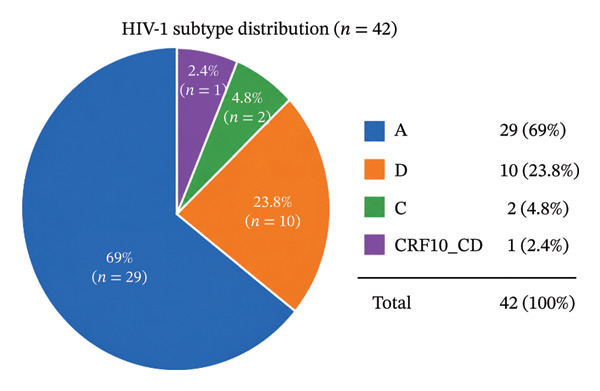
HIV‐1 subtype distribution.

The phylogenetic analysis of the 42 sequences generated from this study revealed that the majority of the HIV‐1 subtypes belonged to Subtype A (69%), followed by Subtype D (23.8%) and Subtype C (4.8%). Circulating recombinant form CRF10_CD (2.4%) was also detected, as presented in Figure 2.

**FIGURE 3:**
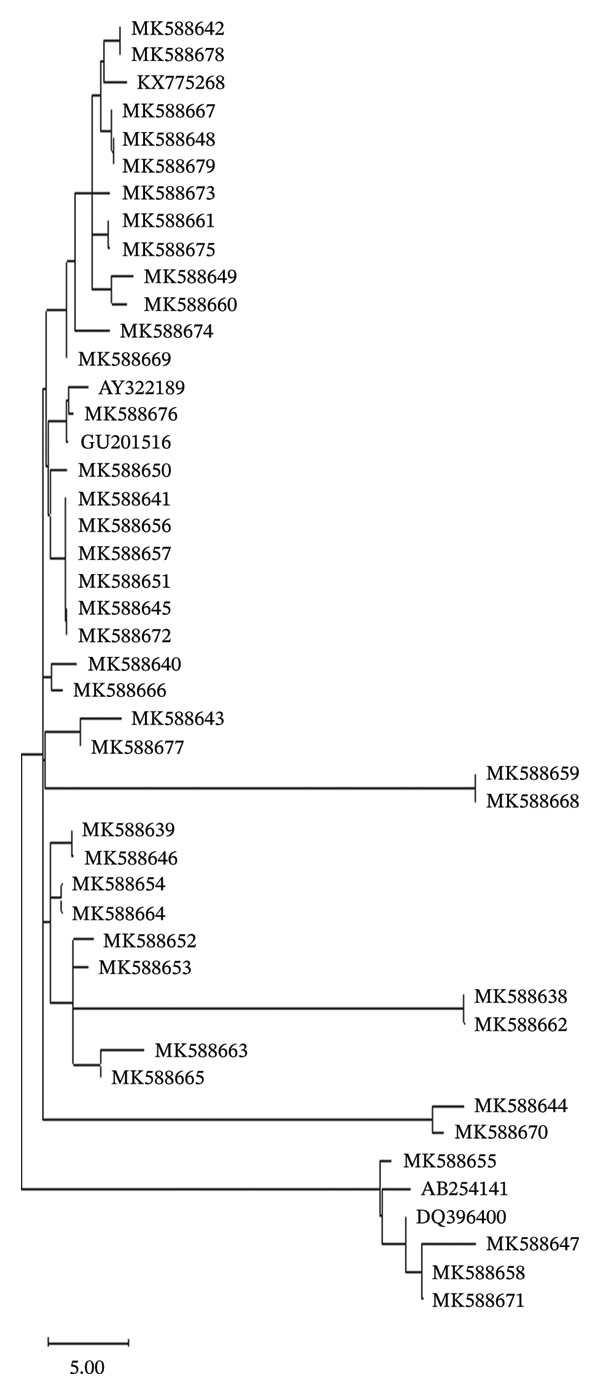
Phylogenetic tree of the HIV‐1 reverse transcriptase sequences from Nairobi County in relation to reference sequences from the Los Alamos HIV database.

Phylogenetic analysis using MEGA 11 software revealed broad genetic diversity among the participants’ HIV‐1 samples, corroborating with reference sequences KX775268, AY322189, GU201515, AB254141, and DQ396400 from the Los Alamos HIV Sequence database, as presented in Figure 3.

## 4. Discussion

This study identified HIV‐1 drug resistance‐associated mutations and circulating viral subtypes among patients receiving ART in Nairobi County, Kenya. Among the successfully sequenced samples, M184V and K103N were the most frequently detected resistance mutations. These mutations have been widely reported in previous Kenyan and regional studies and are commonly associated with resistance to lamivudine, efavirenz, and nevirapine [12].

The predominance of the M184V mutation may reflect selective drug pressure from widespread use of lamivudine‐containing regimens. Although M184V reduces viral fitness, it may compromise the effectiveness of commonly used NRTIs. Similarly, K103N is associated with high‐level resistance to first‐generation NNRTIs and may contribute to treatment failure where these drugs remain in use.

The phylogenetic findings demonstrated substantial HIV‐1 genetic diversity, with subtype A remaining the predominant circulating subtype, consistent with previous studies conducted in Kenya [13, 14]. The detection of CRF10_CD further highlights ongoing viral recombination and the dynamic nature of the HIV epidemic within the region. HIV‐1’s ability to maintain genetic variability offers a survival advantage against the host’s immune system and antiretroviral drugs. This variability results from high replication rates, errors, and recombination during reverse transcription [15, 16]. Understanding this diversity is crucial for developing optimal antiviral regimens and managing treatment, as it allows the virus to evade the host immune system [5].

Importantly, only 42 of the 227 collected samples were successfully amplified and sequenced. This limited number of sequences substantially restricts the statistical strength and generalizability of the findings. The high proportion of nonamplified samples was likely related to low viral loads among participants receiving ART, which may indicate effective viral suppression in a large proportion of the cohort. Consequently, the resistance patterns described in this study should be interpreted cautiously and considered descriptive rather than representative of all patients receiving ART in Nairobi County.

Nevertheless, the findings provide useful preliminary molecular surveillance data regarding HIV‐1 resistance mutations and subtype distribution among treated patients in Nairobi. Continued surveillance involving larger sample sizes and broader genomic characterization would strengthen understanding of evolving resistance patterns and support evidence‐based HIV treatment strategies in Kenya.

## 5. Conclusion

This study identified several HIV‐1 drug resistance‐associated mutations among successfully sequenced samples from patients receiving ART in Nairobi County, with M184V and K103N being the most frequently detected mutations. Subtype A remained the predominant circulating HIV‐1 subtype.

However, the relatively small number of successfully sequenced samples limits the representativeness and broader generalizability of the findings. Therefore, the results should be interpreted cautiously as descriptive observations from a subset of participants.

Despite these limitations, the study contributes additional information on HIV‐1 genetic diversity and resistance‐associated mutations among patients receiving ART in Nairobi County. Further studies involving larger sample sizes and broader molecular surveillance are warranted to better characterize evolving resistance trends and support HIV treatment monitoring efforts in Kenya.

## Author Contributions

Erickson Angiro conceived and designed the study, performed the laboratory experiments, analyzed the data, and drafted the manuscript. Musa Ngayo and Anthony Nyamache supervised the laboratory experiments, performed data analysis, and critically reviewed the manuscript. Caroline Wahome and Juster Mungiria reviewed the manuscript.

## Funding

This study received no specific funding.

## Disclosure

All authors read and approved the final manuscript.

## Conflicts of Interest

The authors declare no conflicts of interest.

## Data Availability

The genome sequences are available in the GenBank database under accession numbers MK588638‐MK588679. The reference sequences were retrieved from the Los Alamos HIV Sequence database under accession numbers KX775268, AY322189, GU201515, AB254141, and DQ396400.
